# Dedifferentiated Liposarcoma in a Giant Esophageal Polyp: A Case Report and Review of the Literature

**DOI:** 10.7759/cureus.4480

**Published:** 2019-04-16

**Authors:** Malav P Parikh, Aswathi Chandran, Jinendra Satiya, Siva Raja, Madhusudhan R Sanaka

**Affiliations:** 1 Gastroenterology and Hepatology, Cleveland Clinic Foundation, Cleveland, USA; 2 Gastroenterology, Hepatology and Nutrition, University of Texas Health Science Center at Houston, Houston, USA; 3 Internal Medicine, University of Miami, John F Kennedy Medical Center, Atlantis, USA; 4 Thoracic Surgery, Cleveland Clinic Foundation, Cleveland, USA

**Keywords:** liposarcoma, fluorescence in situ hybridization, endoscopic release, neoplasia, esophageal cancer

## Abstract

Soft tissue sarcomas represent an extremely rare cause of esophageal masses, and undifferentiated sarcomas are rarer. The proportion of dedifferentiated liposarcomas (DDL) is even lower. The case of a 58-year-old male who complained of dysphagia and was found to have an 18-centimeter long esophageal mass/polyp on esophagogastroduodenoscopy is presented. The lesion was resected endoscopically and a diagnosis of DDL was confirmed by fluorescence in situ hybridization. Due to its rarity, the treatment experience with esophageal DDLs is limited. However, based on our experience, endoscopic resection of the lesion can be considered as the treatment of choice when feasible. We performed a review of the literature to identify and analyze similar reported cases.

## Introduction

Tumors of mesenchymal origin are rare in the gastrointestinal tract, with liposarcoma having an incidence of 0.1% to 5.8 % at autopsy [1]. It is particularly rare in the esophagus, accounting for 1.2% to 1.5 % of all gastrointestinal liposarcomas [2-3]. An even smaller proportion of these tumors represent dedifferentiated liposarcomas (DDL) [4]. According to a study from surveillance, epidemiology, and end results program (SEER) data, the incidence of DDLs, regardless of the primary site, was only 0.64% (171 out of 26,758 cases) of all the soft tissue sarcomas [5]. Given such a rare incidence, the diagnosis may not be suspected initially and, consequently, there are limited data on the effective management of these uncommon esophageal tumors. Esophageal liposarcomas were initially managed by surgical resection, however, with recent advances in technology, endoscopic resection can be an alternative that offers a minimally invasive treatment option [6-8]. We describe a rare case of an esophageal DDL with a review of the literature of similar cases.

## Case presentation

**Figure 1:**
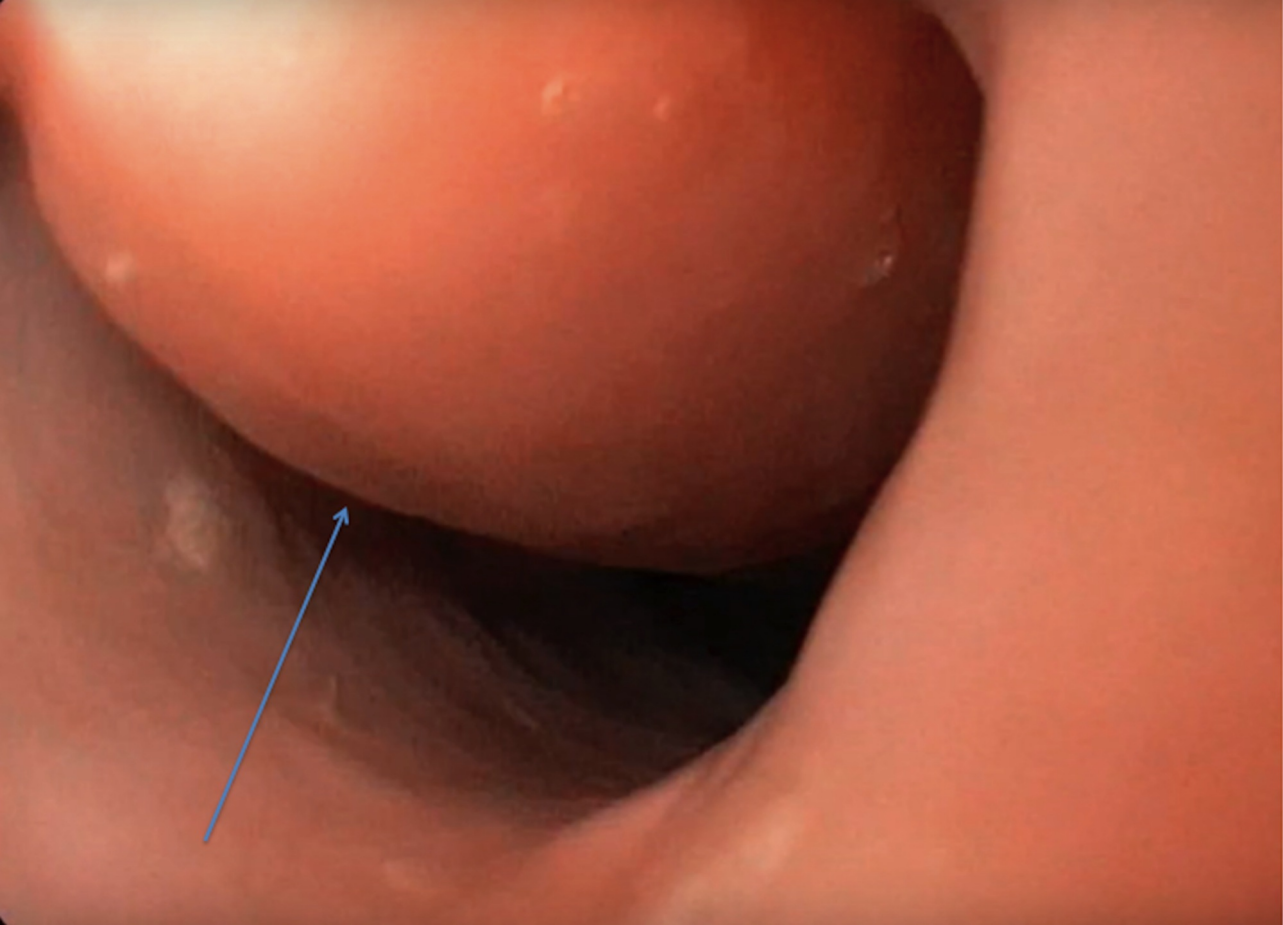
Giant esophageal polyp originating near the cricopharyngeus

A 58-year-old male was evaluated at an outpatient clinic for difficulty in swallowing for the last few months. He complained of dysphagia with solid food but faced no problem with a liquid diet. The patient denied any odynophagia, vomiting, symptoms of gastroesophageal reflux, or weight loss. He did not have any significant past medical history and did not smoke or drink alcohol. Upper esophagogastroduodenoscopy (EGD) was performed for further assessment, which showed, a giant, pedunculated mass arising from the esophageal wall at 18 centimeters (cms) from the incisors, near the cricopharyngeus and extended up to 36 cms from the incisors (Figure 1).

The lesion was partially obstructing and not circumferential. The biopsy was negative for malignancy, and it was suspected to be a giant fibrovascular esophageal polyp. The patient was referred to our center for further investigation and management. We performed a computerized tomography (CT) scan of the chest, which showed severe esophageal dilation, measuring up to 5.4 cms at mid-mediastinal level, with retained food debris. After a multidisciplinary discussion between gastroenterology and the thoracic surgery team, a decision was made to endoscopically resect the mass.

**Figure 2:**
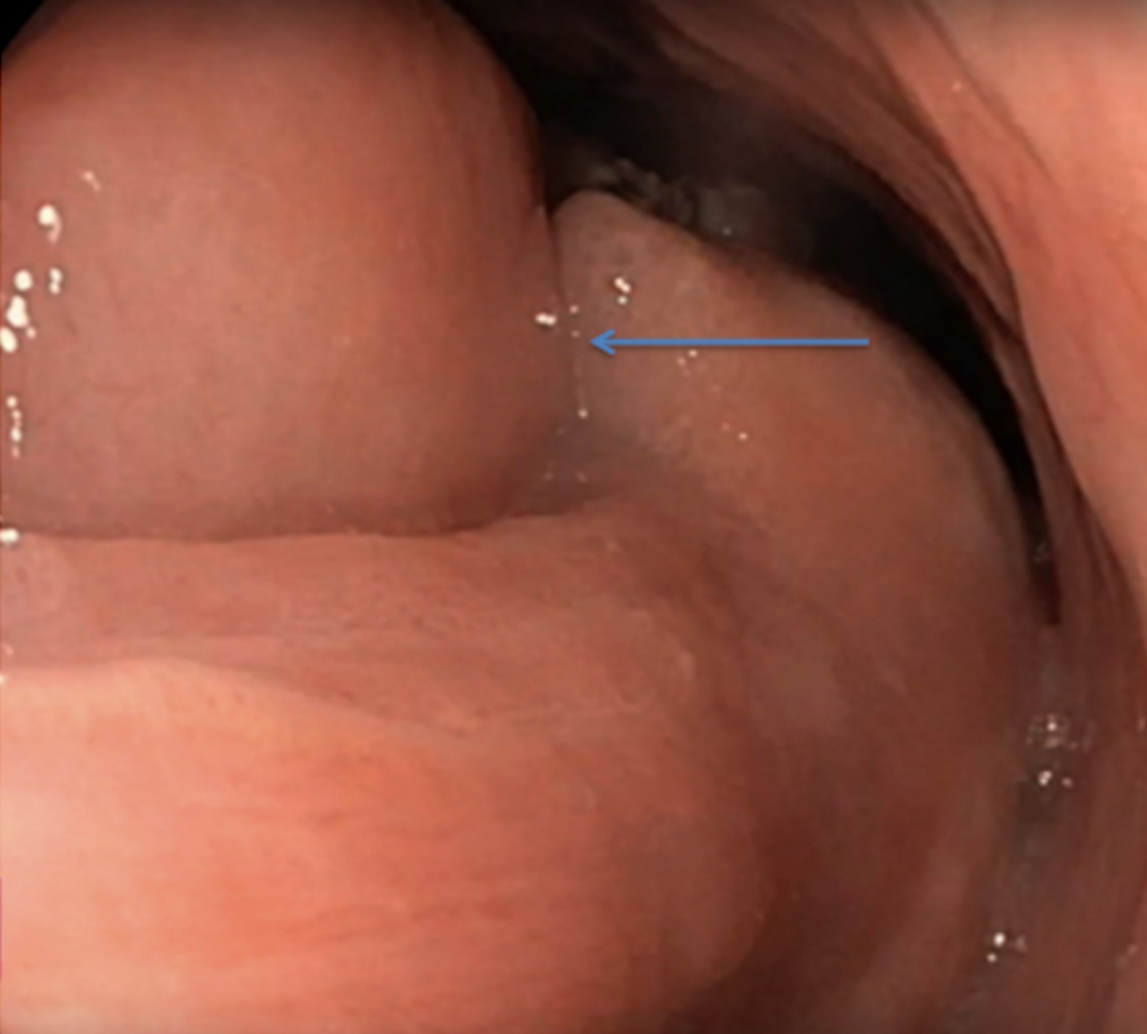
Mid portion of the lesion with a large multi-lobulated mass

During endoscopy, the mid-portion of the lesion was noted to have a large multi-lobulated mass (Figure 2).

**Figure 3:**
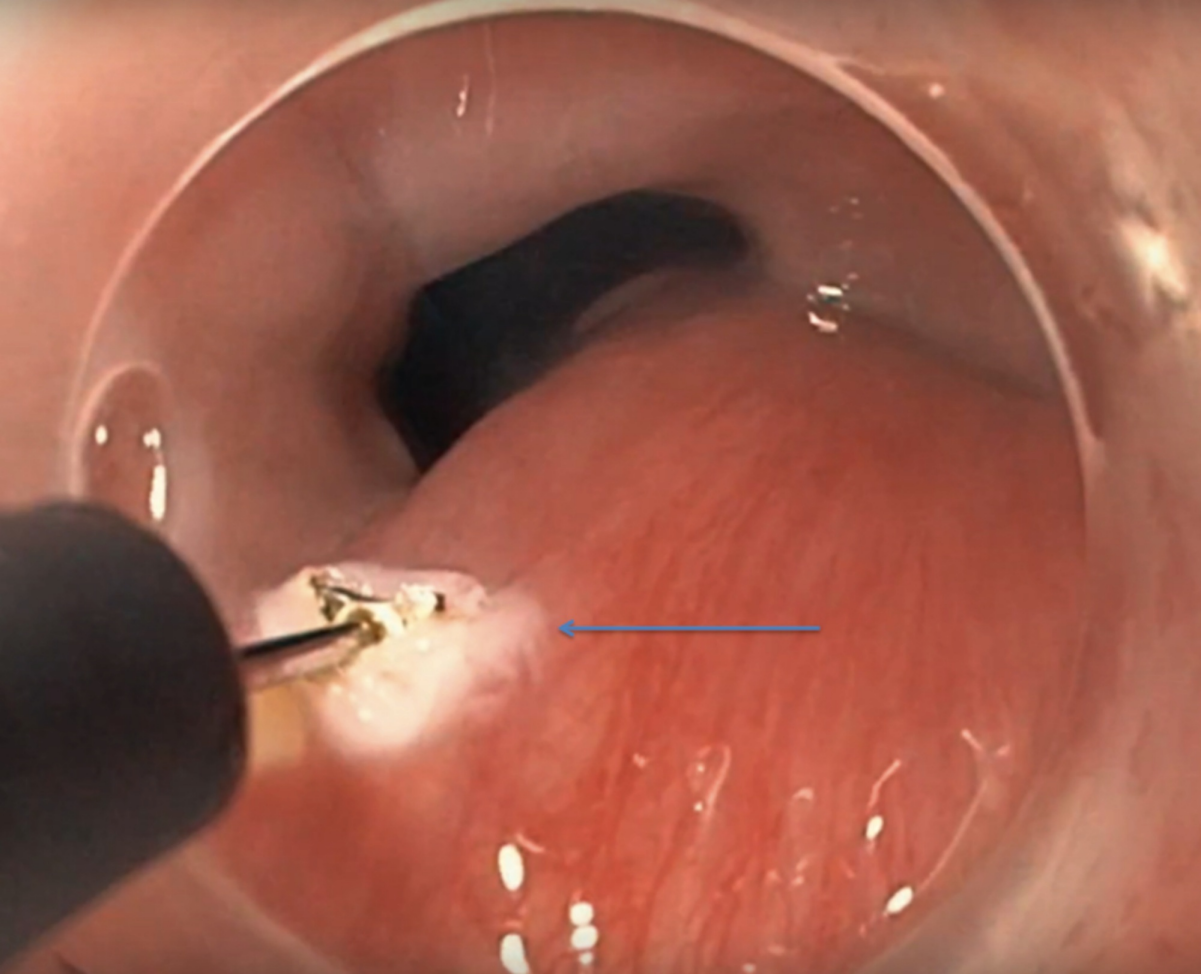
Endoscopic resection of the lesion at the tumor base

At this point, a snare was used to attempt to resect the base; however, due to the presence of the mid lesional, multi-lobulated mass, we were unable to get around it; therefore, electrocautery was used in combination with an IT knife (KD-611L, Olympus America, PA, US) to transect the lesion at the base (Figure 3).

**Figure 4:**
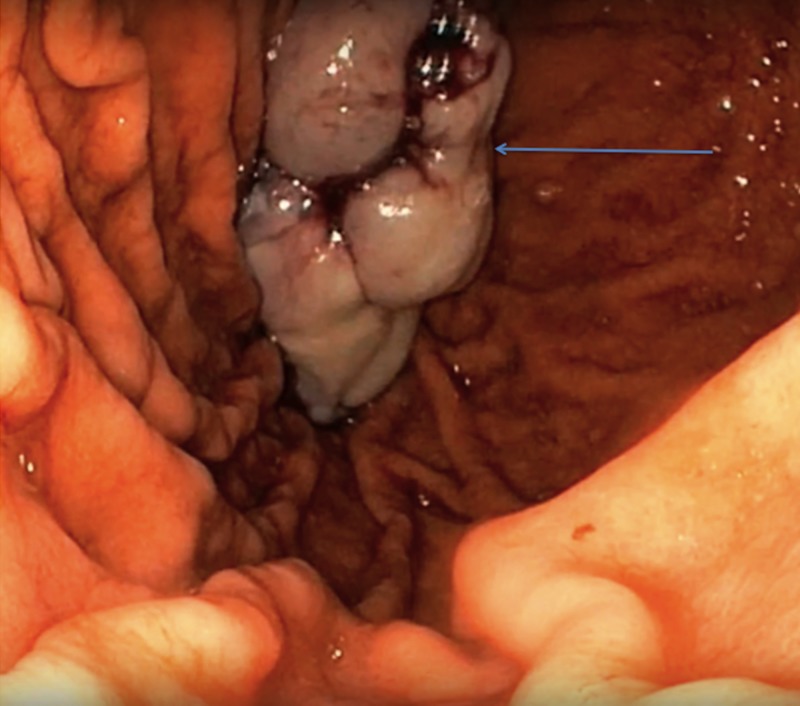
Resected esophageal mass in the stomach

At the completion of this, there was adequate hemostasis at the base and an endo clinch was used to further reinforce it. The entire mass was attempted to be pulled out of the esophagus but could not be moved out past the upper esophageal sphincter. Therefore, it was pushed back into the stomach, cut into smaller pieces using a hot snare, and removed in a piecemeal fashion (Figure 4).

The specimen was sent for routine pathological evaluation. A barium esophagram performed on the first postoperative day showed no evidence of a leak, and the patient tolerated a clear liquid diet without any complications. The pathology report revealed a diagnosis of DDL. The patient was advised about further imaging with positron emission tomography (PET)-CT scan and referral to a radiation oncologist, but he refused additional treatment.

## Discussion

Dedifferentiated liposarcoma (DDL) is an extremely rare cause of an esophageal mass, with only eight reported cases so far. We did a literature review to identify the demographic details, clinical presentation, histology, treatment, and follow-up of esophageal DDLs (Table 1).

**Table 1:** Demographics, clinical presentation, histology, treatment, and follow-up of dedifferentiated liposarcoma (DDL) of the esophagus CPM: Carboxypeptidase M; DDL: Dedifferentiated liposarcoma; MDM2: Mouse double minute 2 homolog; +: Positive

Author	Age	Sex	Symptoms	Histology	Treatment	Follow-up
Brett et al. [6]	75	M	Dysphagia	Marked cytological atypia. + CPM	Endoscopic resection	No recurrence at 20 months
Torres-Mora et al. [7]	81	M	Dysphagia	Spindle cell sarcoma. + CPM	Endoscopic resection	Close endoscopic follow-up
Will et al. [9]	60	M	Dysphagia	DDL	Endoscopic resection	No endoscopic recurrence at 1 year
Graham et al. [10]	67,42,75,66	M,M,M,F	Dysphagia	DDL. + MDM2	-	Two alive at 29 months

All patients were males, with an average age of 75 years (50-81 years) and the most common symptom was dysphagia followed by weight loss. Successful endoscopic resection was done in three of five (60%) and surgical resection in two of five (40%) of the cases.

Five pathologic subtypes of liposarcoma are described in the literature, including well-differentiated, dedifferentiated, myxoid, pleomorphic, and round. DDL is defined as a tumor containing an atypical lipomatous tumor or a well-differentiated liposarcoma admixed to a component of high-grade non-lipogenic sarcoma. The genetic hallmark of these tumors is supernumerary ring chromosomes that contain amplification of chromosome 12q13-15. The MDM2 (mouse double minute 2 homolog) gene is present in this region of chromosome 12 [9]. The CPM (carboxypeptidase M) gene is telomeric to the MDM2 locus on chromosome 12q15 and is consistently co-amplified with MDM2, allowing its use as a surrogate to MDM2. It is important to perform a careful morphological study and MDM2 amplification by fluorescence in situ hybridization (FISH) to identify liposarcomas before diagnosing a patient with a giant fibrovascular polyp [10].

**Figure 5:**
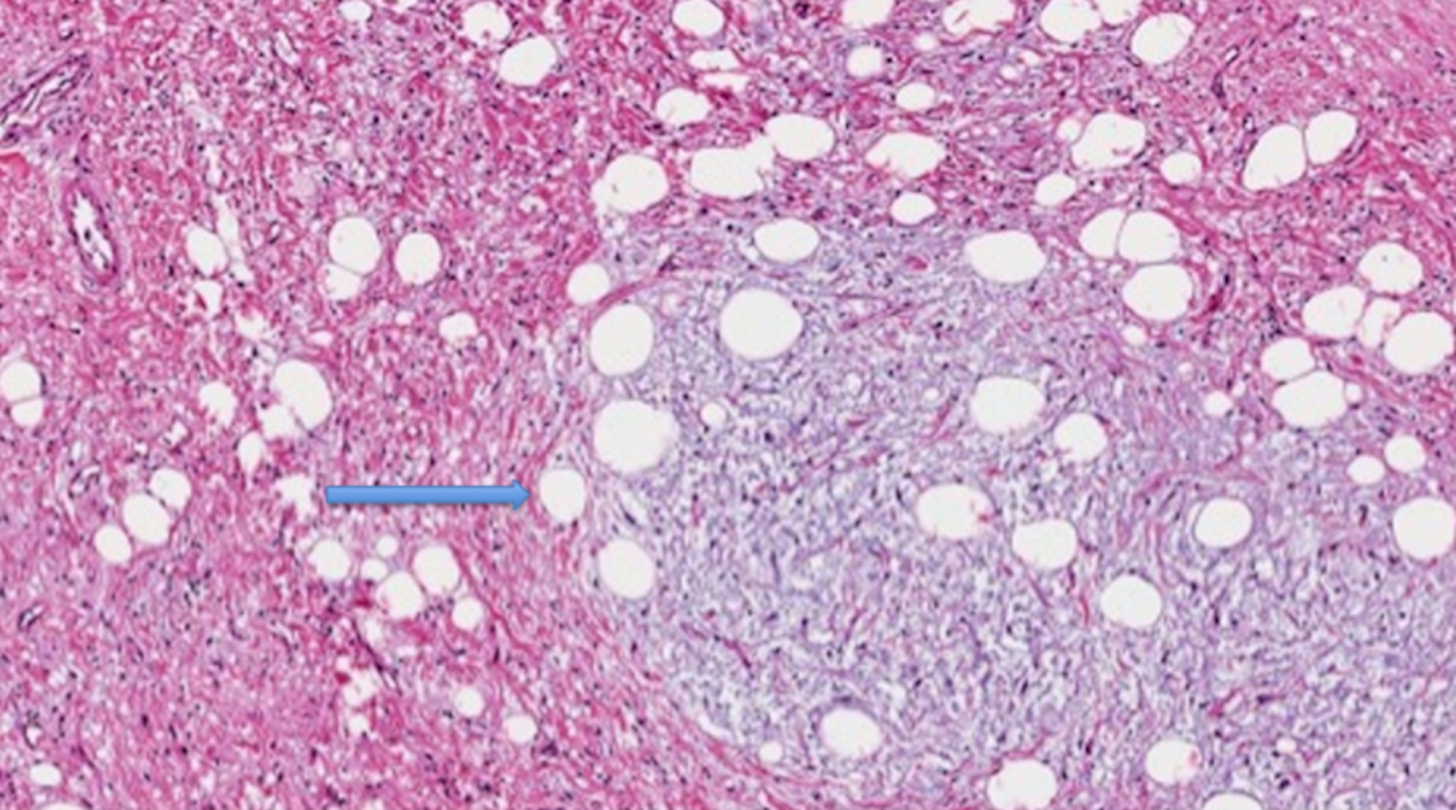
Well-differentiated liposarcoma component characterized by adipocytes, myxoid change, and scattered atypical cells

In our case, the histology of the resected specimen showed both well-differentiated and dedifferentiated components. The well-differentiated part was characterized by adipocytes, myxoid change, and scattered atypical cells (Figure 5).

**Figure 6:**
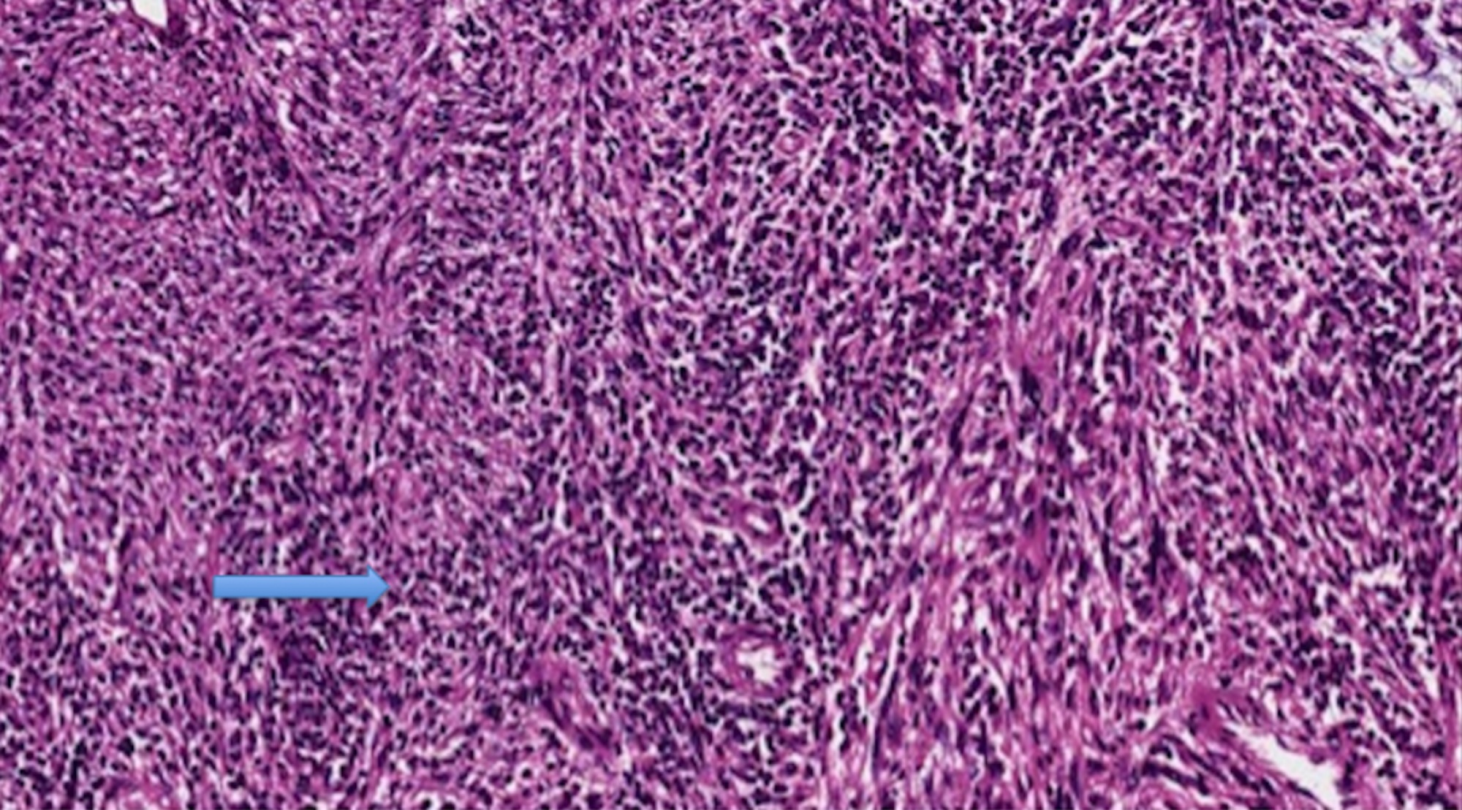
Dedifferentiated component with fascicles of hyper-cellular spindle cells without lipogenic differentiation

The DDL component consisted of fascicles of hyper-cellular spindle cells without lipogenic differentiation (Figure 6). 

Immunohistochemical stain was performed to further explore the presence of DDL. The majority of the tumor cells displayed at least one centromeric chromosome 12 signal and many MDM2 signals, with an MDM2/CEP12 ratio >/= 2.0, consistent with the amplification of MDM2. The interphase fluorescence in situ hybridization (FISH) technique was used to confirm the amplification of the MDM2 gene locus and it was positive. The overall histologic and molecular findings supported the diagnosis of esophageal DDL.

Due to its rarity, the treatment experience with esophageal DDLs is limited. A study by Wu et al. from the SEER registry demonstrated that patients with esophageal sarcomas were more likely to have localized disease, to be treated with surgery, and to have better overall survival (37 vs.14 %; p < 0.0001) than patients with esophageal carcinoma. The authors also proposed that surgery should be the primary treatment for patients with esophageal sarcoma due to the survival benefit [11]. A review of eight cases of pleomorphic sarcoma of the esophagus demonstrated a favorable response to radical resection of the tumor [12-13]. Riva et al. described a case of an 81-year-old male with DDL who underwent lateral pharyngotomy and resection through a cervical incision, with no endoscopic recurrence at one year [13]. Based on our literature review and extrapolation from the above results, surgical or endoscopic resection can be considered as the treatment of choice for esophageal DDLs. Further studies are required to establish the potential roles of chemotherapy and radiotherapy.

Previously esophageal liposarcomas were treated by surgical methods, including simple enucleation and partial or total esophagectomy. However, this approach is more invasive with a longer postoperative recovery period, hospital stay, and cost as compared to endoscopic resection. This case demonstrates that even giant esophageal DDLs can be successfully resected by the endoscopic technique. Even though there are no guidelines on the duration of follow-up, esophageal liposarcomas can recur with a latency period of up to 25 years after resection [14]. Most soft tissue sarcomas recur within the first five years after treatment. The theories used to explain recurrences include incomplete resection, development of a new primary tumor, and genetic susceptibility. The risk of recurrence depends on histological grade and subtype, size of the primary tumor, adequate surgery, and time from initial treatment.

Recommendations for surveillance include annual upper endoscopy with biopsies and rigorous clinical follow-up with history and physical examinations performed every three months to identify those with new-onset dysphagia [15]. A chest X-ray is helpful in identifying asymptomatic lung metastases. A suspicious nodule identified on chest X-ray warrants further evaluation with a computed tomography (CT) scan. Follow-up can be extended to every six months after the first three years. Annual visits are acceptable thereafter. It is important to educate patients about the possibility of late relapse and ensure enrollment in long-term follow-up programs with close endoscopic and radiographic surveillance.

## Conclusions

Esophageal DDLs are rare tumors but should be considered as a differential diagnosis when evaluating esophageal masses/polyps. FISH showing the amplification of the MDM2 gene locus is diagnostic. Resection of the esophageal DDL can be considered as the treatment of choice and with recent advances in technology, endoscopic resection of such tumors is achievable, offering several potential benefits over surgical resection.
